# The impact of retinal fluid tolerance on the outcomes of neovascular age-related macular degeneration treated using aflibercept: A real-world study

**DOI:** 10.1371/journal.pone.0271999

**Published:** 2022-07-28

**Authors:** Yu-Ting Jeng, Tso-Ting Lai, Chao-Wen Lin, Ta-Ching Chen, Yi-Ting Hsieh, Chang-Ping Lin, Tzyy-Chang Ho, Chung-May Yang, Chang-Hao Yang

**Affiliations:** 1 Department of Ophthalmology, National Taiwan University Hospital, Taipei, Taiwan; 2 Graduate Institute of Clinical Medicine, College of Medicine, National Taiwan University, Taipei, Taiwan; 3 Department of Ophthalmology, College of Medicine, National Taiwan University, Taipei, Taiwan; National Yang-Ming University Hospital, TAIWAN

## Abstract

This study investigated the impact of retinal fluid tolerance on retinal thickness and visual acuity in patients with neovascular age-related macular degeneration after 18 months of treatment using intravitreal aflibercept. This retrospective study was based on the medical records of 90 eyes presenting persistent or recurrent retinal fluid retention after 3 months of aflibercept loading injections. We defined the fluid tolerance ratio as the sum of fluid-tolerance duration divided by the total duration of retinal fluid observed throughout the follow-up period. Eyes were categorized into strict, intermediate, and relaxed group based on their fluid tolerance ratio (= 0, <30%, > = 30%, respectively). The mean total follow-up time was 556 days. The relaxed group required fewer injections than the strict group (4.92 vs 7.50 injections, P < 0.01) and presented a similar reduction in retinal thickness (-57.50 vs -71.65 μm, P = 0.83). Nonetheless, the two groups were similar in terms of final visual acuity (logarithm of the minimum angle of resolution 0.72 vs 0.70, P = 0.95) and visual gains (4.21 vs -1.12 letters, P = 0.56). These results indicate that in the setting of limited medical resources, a fluid-tolerant approach provides comparable gains in visual acuity. Reducing the number of injections may also improve adherence to therapy.

## Introduction

Neovascular age-related macular degeneration (nAMD) is a common cause of visual impairment among the elderly [1]. The advent of intravitreal anti-vascular endothelial growth factor (anti-VEGF) therapy has greatly improved the treatment outcomes of patients with nAMD [2,3]. The VIEW 1 and VIEW 2 studies demonstrated that aflibercept treatment at a fixed dosing interval can improve vision, with a mean visual gain of 8.4 to 9.3 letters after 1 year [3]. The treatment burden of fixed-interval dosing has prompted the development of alternative treatment strategies, such as treat-and-extend (T&E) or pro re nata (PRN), to reduce the number of injections without compromising visual gains.

In the TREX-AMD and TREND studies, the presence of retinal fluid was assumed to be an indicator of disease activity which required treatment, therefore treatment extension was only allowed when all retinal fluids were eliminated [4,5]. However, evidence suggests that there is no need to entirely dry the retina to achieve the desired visual outcome. In *post hoc* analysis of the CATT study, no significant differences in final visual outcomes were observed between the PRN-injection group and patients receiving monthly injections aimed at completely drying the retina [6]. The FLUID study also determined that tolerance for a certain degree of subretinal fluid did not compromise final visual outcomes [7]. The presence of anti-VEFG-resistant subretinal fluid has also been shown to be associated with reduced incidence of macular atrophy [8,9]. Note that relatively few studies have addressed the impact of fluid-tolerance on the treatment outcomes of nAMD in the real world. Our primary objective in the current study was to determine whether fluid tolerance in the implementation of intravitreal aflibercept treatment affects anatomical or functional outcomes in nAMD patients presenting recurrent or persistent retinal fluid.

## Materials and methods

### Study group and outcomes

This was a single center, retrospective study conducted at National Taiwan University Hospital. Data analysis began with all patients who received National Health Insurance (NHI) coverage for intravitreal aflibercept injection for nAMD between January 2016 and July 2019. In accordance with NHI regulations, all enrolled patients were aged 50 years or older, had best-corrected visual acuity between 20/40 to 20/400 at baseline, and presented active nAMD lesions in fluorescein angiography and optical coherence tomography (OCT) images. Patients were eligible to receive self-paid intravitreal anti-VEGF injection in cases where the above criteria were not met, and could apply for NHI covered treatment in cases where the criteria were met. This study was conducted in adherence to the tenets of the Declaration of Helsinki. This study was approved by the National Taiwan University Hospital Research Ethics Committee (case number: 202006010RIFC).

All patients were treated under the discretion of the attending clinicians, and most of the cases involved the extended PRN regimen, where the follow-up intervals were not strictly fixed. Each patient was examined from the day of their first injection until the 18th month after the first injection. Patients with follow-up durations of less than 18 months were excluded. To investigate the effect of fluid tolerance, we excluded patients presenting dry retina at every follow-up visit after 3 monthly loading injections. Only patients with persistent or recurrent retinal fluid from month 3 to month 18 were included. Eyes receiving intravitreal anti-VEGF injection within 1 year prior to the first injection were excluded. Patients with polypoidal choroidal vasculopathy, epiretinal membrane, diabetic retinopathy, or other maculopathies other than nAMD were excluded. We also excluded eyes receiving prior verteporfin photodynamic therapy. If two eyes of the same patient met the inclusion criteria, only the worse eye was included.

The following data were extracted from the patient’s medical records: demographic data, findings on OCT, fluorescein angiography, and best-corrected visual acuity (BCVA) at baseline and at each visit. Patients with any missing data at month 3, 6, 12, and 18 were excluded. Morphological data were reviewed and interpretated by two authors (Y.T.J. and C.H.Y.). CNV pattern was determined by OCT features and fluorescein angiography. Pachychoroid was defined as choroidal thickness exceeding 300 μm within a 3 mm × 3 mm region of an OCT scan centered at the fovea. Retinal fluid was classified as IRF or SRF. Fluid tolerance was defined as not receiving anti-VEGF injection despite the presence of retinal fluid noted during a clinical visit. For example, if an anti-VEGF injection was not administered despite the presence of retinal fluid during visit A, then the length of the interval between visit A and the next clinical appointment (visit B) was deemed the fluid-tolerance duration. We defined the fluid tolerance ratio as the summed duration of the fluid-tolerance periods divided by the total duration of fluid retention observed throughout the entire follow-up period. Eyes were categorized according to fluid tolerance ratio during the 18-month follow up as follows: strict (zero tolerance), intermediate (tolerance ratio of less than 30%), and relaxed (tolerance ratio of 30% or greater). Dry retina was defined as the absence of both SRF and IRF. The primary outcomes included changes in BCVA and central retinal thickness (CRT) at month 3, 6, 12, and 18. Retinal fluids were included in the calculation of CRT.

### Statistical analysis

A paired sample t-test was used to detect differences in the mean CRT and BCVA before and after treatment. The chi-squared test and Fisher’s exact test were used where appropriate to compare differences between the three fluid tolerance groups in terms of categorical variables. Monte Carlo simulation was applied for Fisher’s exact test. Analysis of variance (ANOVA) was used to compare differences in continuous variables. Welch’s ANOVA was applied in cases where intergroup heterogeneity of variance was significant. Post hoc analysis was performed using the Bonferroni correction. Both univariate and multivariate linear regression was used to identify factors associated with baseline visual acuity and changes in visual acuity. In all analysis, statistical significance was defined as a *P* value of less than 0.05. Data analysis was conducted using Statistical Product and Service Solutions Version 25.0 (IBM Corp. IBM SPSS Statistics for Windows).

## Results

### Study group and baseline characteristics

A total of 90 eyes (90 patients) were included in our final analysis. Regarding their fluid tolerance ratio during follow-up, 20 cases were categorized as strict, 22 cases were intermediate, and 48 cases were relaxed. Baseline demographic data and clinical characteristics are listed in Table 1. The mean age of the patients was 72.7±10.4 years, and 50 patients were male (55.6%). The mean total follow-up time was 556 days (median 553 days, standard deviation 25 days). Mean overall clinical visits from 3 months to 18 months were 7.99±1.23. The average logarithm of the minimum angle of resolution (logMAR) was as follows: strict group (0.67), intermediate group (0.83), and relaxed group (0.80) (*P* = 0.50). We did not observe significant intergroup differences in the proportion of patients presenting baseline BCVA of not worse than 20/40 (*P* = 0.16) or not better than 20/200 (*P* = 0.97). Classic choroidal neovascularization (CNV) pattern was detected in 61 eyes (67.8%) in our study population, with no significant intergroup differences (*P* = 0.71). No retinal angiomatous proliferation lesion was noted in our study population. We did not observe significant intergroup differences in terms of retinal fluid component; i.e., most of the patients in all the three groups presented “subretinal fluid (SRF) only”. The mean CRT was higher in the intermediate group (321.8±81.7 μm) than in the strict group (309.2±69.0 μm) and the relaxed group (300.4±90.4 μm) but without statistical significance (*P* = 0.61). We did not observe significant intergroup differences in the proportion of patients presenting pigment epithelial detachment (PED), or pachychoroid.

**Table 1:** Baseline demographic data and clinical characteristics.

	Strict (N = 20)	Intermediate (N = 22)	Relaxed (N = 48)	Total (N = 90)	P value
Age, yrs, mean ± SD	72.7 ± 10.4	70.5 ± 11.2	71.2 ± 9.0	71.3 ± 9.8	0.77
Sex, no. (%)					0.82
Male	10 (50.0)	12 (54.5)	28 (58.3)	50 (55.6)	
Female	10 (50.0)	10 (45.5)	20 (41.7)	40 (44.4)	
BCVA					
LogMAR, mean ± SD	0.67 ± 0.43	0.83 ± 0.49	0.80 ± 0.47	0.78 ± 0.46	0.50
BCVA ≧ 20/40, no. (%)	6 (30.0)	2 (9.1)	7 (14.6)	15 (16.7)	0.16
BCVA ≦ 20/200, no. (%)	7 (35.0)	8 (36.4)	16 (33.3)	31 (34.4)	0.97
CNV type, no., (%)					0.71
Classic	15 (75.0)	14 (63.6)	32 (66.7)	61 (67.8)	
Occult	5 (25.0)	8 (36.4)	16 (33.3)	29 (32.2)	
Retinal fluid components, no. (%)					0.06
IRF only	0 (0.0)	3 (13.6)	11 (22.9)	14 (15.6)	
SRF only	16 (80.0)	11 (50.0)	28 (58.3)	55 (61.1)	
Combined SRF and IRF	4 (20.0)	8 (36.4)	9 (18.8)	21 (23.3)	
CRT, um, mean ± SD	309.2 ± 69.0	321.8 ± 81.7	300.4 ± 90.4	307.5 ± 83.6	0.61
PED, no. (%)	12 (60.0)	13 (59.1)	23 (47.9)	48 (53.3)	0.54
Pachychoroid, no. (%)	2 (10.0)	2 (9.1)	3 (6.3)	7 (7.8)	0.77
Clinical visits from 3 to 18 months, no., mean ± SD	7.95 ± 1.15	8.23 ± 1.07	7.90 ± 1.34	7.99 ± 1.23	0.58

BCVA, best-corrected visual acuity; CRT, central retinal thickness; CNV, choroidal neovascularization; IRF, intraretinal fluid; LogMAR, logarithmic minimum angle of resolution; PED, pigment epithelial detachment; SD, standard deviation; SRF, subretinal fluid.

### Factors associated with baseline visual acuity

Table 2 presents regression analysis of factors associated with baseline LogMAR. In univariate analysis, baseline CRT and presence of IRF at baseline were both inversely correlated with baseline visual acuity (both *P* < 0.01). After adjusting for age, sex, and baseline CRT, both baseline CRT and presence of IRF at baseline remained significant correlation with poorer baseline visual acuity. The factors presenting a non-significant correlation with baseline visual acuity included the following: age, sex, SRF at baseline, PED at baseline, and pachychoroid at baseline.

**Table 2:** Regression analysis of factors associated with baseline LogMAR.

	Univariate coefficient	P value	Adjusted coefficient^¶^	P value
Age	0.036	0.74	0.068	0.51
Sex (male = 1)	0.099	0.35	0.097	0.35
Baseline CRT, μm	0.310	<0.01	0.317	<0.01
SRF at baseline	-0.020	0.85	-0.075	0.48
IRF at baseline	0.338	<0.01	0.271	0.02
PED at baseline	-0.089	0.40	-0.088	0.40
Pachychoroid at baseline	0.016	0.88	-0.025	0.81

CRT, central retinal thickness; IRF, intraretinal fluid; LogMAR, logarithmic minimum angle of resolution; PED, pigment epithelial detachment; SRF, subretinal fluid.

^¶^Age, sex, and baseline CRT were used for adjustment in multivariate analysis.

### Fluid tolerance and the number of injections

As shown in Table 3, the fluid tolerance levels were as follows: strict group (0.0± 0.0%), intermediate group (19.4±6.5%), and relaxed group (50.8±14.0%). The duration of fluid tolerance divided by total follow up time was as follows: strict group (0.0± 0.0%), intermediate group (13.6±6.3%), and relaxed group (38.0±16.0%). The duration of SRF tolerance divided by total follow up time was 15.7±19.1%, and the ratio was 11.0±17.2% in terms of Intraretinal fluid (IRF) tolerance. Throughout the follow-up period, the total duration of retinal fluid was as follows: strict group (53.7±23.2%), intermediate group (71.4±21.8%), and relaxed group (74.9±23.7%; *P* < 0.01). In the overall study population, the total average number of aflibercept injections was 5.99±2.24. The average number of injections at month 18 was as follows: relaxed group (4.92±1.69), intermediate group (6.95±2.15) and strict group (7.50±2.24; *P* < 0.01). The average interval between injections was longest in the relaxed group and shortest in the strict group (*P* < 0.01), and the results remained true after adjusted for the total duration of retinal fluid (*P* < 0.01). The overall incidence of macular atrophy was 10.0% at 18 months, with no significant intergroup differences (*P* > 0.99).

**Table 3:** Fluid tolerance and injection intervals among strict, intermediate, and relaxed groups.

	Strict (N = 20)	Intermediate (N = 22)	Relaxed (N = 48)	Total (N = 90)	P value
Fluid tolerance ratio, %, mean ± SD	0.0 ± 0.0	19.4 ± 6.5	50.8 ± 14.0	31.9 ± 24.0	-
Duration of fluid tolerance/total follow up time, %, mean ± SD					
SRF	0.0 ± 0.0	7.1 ± 8.8	26.1 ± 20.1	15.7 ± 19.1	-
IRF	0.0 ± 0.0	6.9 ± 7.6	17.5 ± 20.7	11.0 ± 17.2	-
SRF or IRF	0.0 ± 0.0	13.6 ± 6.3	38.0 ± 16.0	23.6 ± 20.2	-
Duration of retinal fluid / total follow up time, %, mean ± SD	53.7 ± 23.2	71.4 ± 21.8	74.9 ± 23.7	69.3 ± 24.4	<0.01
Injection number, no., mean ± SD					
Month 3	2.50 ± 0.61	2.36 ± 0.73	2.17 ± 0.88	2.29 ± 0.80	0.26
Month 6	3.55 ± 1.05	3.14 ± 0.83	2.79 ± 1.13	3.04 ± 1.08	0.03
Month 12	5.55 ± 1.79	4.91 ± 1.31	3.90 ± 1.45	4.51 ± 1.64	<0.01
Month 18	7.50 ± 2.24	6.95 ± 2.15	4.92 ± 1.69	5.99 ± 2.24	<0.01
Average injection interval of total follow-up time, wks, mean ± SD	11.7 ± 4.0	12.4 ± 3.7	18.8 ± 10.4	15.6 ± 8.5	<0.01
Average injection interval in the presence of retinal fluid, wks, mean ± SD	7.1 ± 1.4	9.4 ± 1.7	14.6 ± 5.0	11.7 ± 5.0	<0.01
Macular atrophy at 18 months, no. (%)	2 (10.0)	2 (9.1)	5 (10.4)	9 (10.0)	>0.99

IRF, intraretinal fluid; SD, standard deviation; SRF, subretinal fluid.

### Changes in CRT over time

Table 4 illustrates the changes in CRT and BCVA at various time points. Compared to baseline CRT values (307.5±83.6 μm), we observed a significant decrease at month 12 (251.3±75.3 μm; *P* < 0.01) and at month 18 (245.6±73.3 μm; *P* < 0.01). The strict group presented the lowest CRT (237.5±54.2 μm) at month 18; however, it was not significantly lower than that of the intermediate group (258.8±86.9 μm) and the relaxed group (242.9±74.1 μm; *P* = 0.60). The strict group also presented the most pronounced CRT reduction at month 18 (-71.7±84.7 μm); however, it was not significantly better than that of the intermediate group (-63.0±105.3 μm) or the relaxed group (-57.5±81.4 μm; *P* = 0.83). In fact, we observed no significant intergroup differences in CRT or changes in CRT at months 3, 6, 12, or 18.

**Table 4:** Central retinal thickness and best-corrected visual acuity changes among strict, intermediate, and relaxed groups.

	Strict (N = 20)	Intermediate (N = 22)	Relaxed (N = 48)	Total (N = 90)	P value
CRT, um, mean ± SD					
Month 0	309.2 ± 69.0	321.8 ± 81.7	300.4 ± 90.4	307.5 ± 83.6	0.61
Month 3	225.1 ± 36.7	243.6 ± 54.9	246.4 ± 77.8	241.0 ± 65.3	0.46
Month 6	259.2 ± 72.2	249.6 ± 60.9	247.8 ± 80.3	250.8 ± 73.6	0.84
Month 12	248.6 ± 68.0	245.1 ± 51.6	255.3 ± 87.4	251.3 ± 75.3	0.86
Month 18	237.5 ± 54.2	258.8 ± 86.9	242.9 ± 74.1	245.6 ± 73.3	0.60
Change in CRT, um, mean ± SD					
Month 3	-84.1 ± 78.6	-78.2 ± 93.9	-53.9 ± 62.7	-66.6 ± 75.2	0.23
Month 6	-50.0 ± 98.9	-72.2 ± 100.1	-52.6 ± 74.6	-56.8 ± 86.4	0.63
Month 12	-60.6 ± 82.3	-76.6 ± 101.0	-45.1 ± 86.9	-56.3 ± 89.5	0.39
Month 18	-71.7 ± 84.7	-63.0 ± 105.3	-57.5 ± 81.4	-62.0 ± 87.6	0.83
LogMAR BCVA, mean ± SD					
Month 0	0.67 ± 0.43	0.83 ± 0.49	0.80 ± 0.47	0.78 ± 0.46	0.50
Month 3	0.65 ± 0.48	0.83 ± 0.56	0.66 ± 0.46	0.70 ± 0.49	0.37
Month 6	0.61 ± 0.53	0.72 ± 0.51	0.67 ± 0.52	0.67 ± 0.51	0.79
Month 12	0.65 ± 0.49	0.68 ± 0.47	0.73 ± 0.50	0.70 ± 0.49	0.81
Month 18	0.70 ± 0.46	0.74 ± 0.45	0.72 ± 0.48	0.72 ± 0.47	0.95
Change in BCVA, letters, mean ± SD					
Month 3	1.08 ± 11.78	0.06 ± 13.54	6.79 ± 15.64	3.88 ± 14.56	0.12
Month 6	3.25 ± 20.84	5.61 ± 10.71	6.37 ± 15.37	5.49 ± 15.69	0.76
Month 12	1.34 ± 17.12	7.51 ± 20.36	3.52 ± 17.39	4.01 ± 18.02	0.53
Month 18	-1.12 ± 17.98	4.58 ± 21.67	4.21 ± 19.84	3.11 ± 19.82	0.56

BCVA, best-corrected visual acuity; CRT, central retinal thickness; SD, standard deviation.

### Changes in retinal fluid components over time

**Fig 1:**
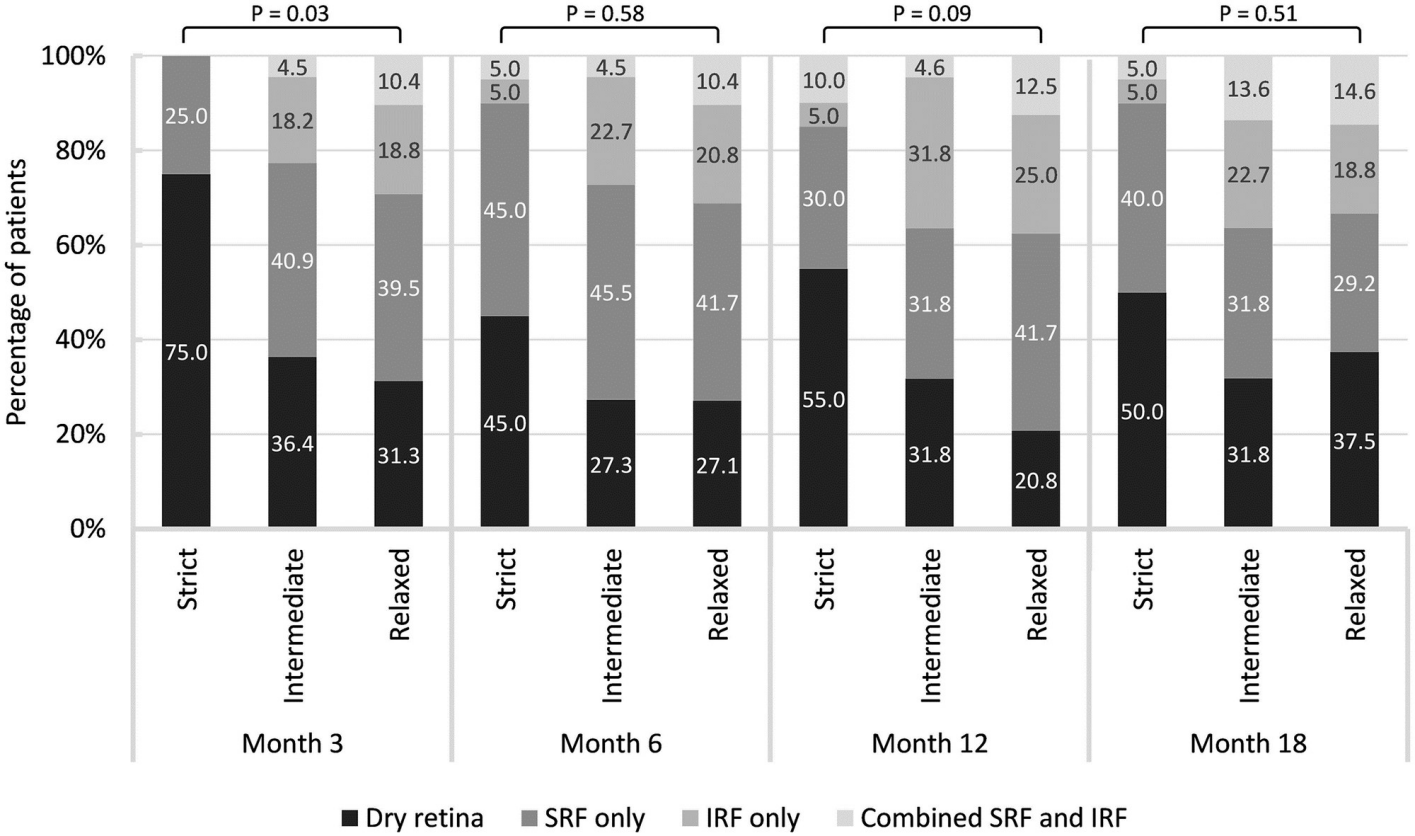
Retinal fluid components among strict, intermediate, and relaxed group at various time points.

Fig 1 illustrates the proportion of patients demonstrating dry retina, SRF only, IRF only, or combined SRF and IRF in each of the groups at various points in time. We observed significant intergroup differences in retinal fluid components at month 3. At that point, 75% of the patients in the strict group achieved dry retina, whereas 63.6% of patients in the intermediate group and 68.7% of patients in the relaxed group presented residual retinal fluid (*P* = 0.03). The relaxed group presented the highest rate of combined SRF and IRF at months 3, 6, 12, and 18. At month 18 in the strict group, the proportion of patients with dry retina dropped to 50.0%, and the proportion of patients with combined SRF and IRF increased to 5.0%. Intergroup differences in the distribution of retinal fluid components did not reach the level of statistical significance at months 6, 12, or 18. Most of the patients with residual retinal fluid presented with “SRF only” in all three groups. Two patients (10%) in the strict group, 5 patients (22.7%) in the intermediate group, and 11 patients (20.0%) in the relaxed group never achieved dry retina during the 18-month follow up (*P* = 0.50).

### BCVA at various time points

**Fig 2:**
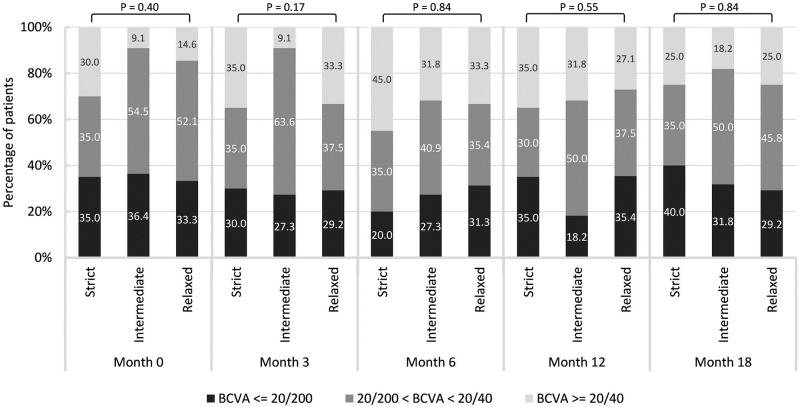
BCVA among strict, intermediate, and relaxed groups at various time points.

Compared to baseline LogMAR BCVA (0.78±0.46), we observed improvements at month 12 (0.70±0.49; *P* = 0.04) and at month 18 (0.72±0.47; *P* = 0.14), as shown in Table 4. At month 18, the mean BCVA were as follows: strict group (0.70±0.46), intermediate group (0.74±0.45), and relaxed group (0.72±0.48) (*P* = 0.95). Likewise, we did not observe significant differences between the three groups in terms of mean BCVA at month 3 (*P* = 0.50), month 6 (*P* = 0.79), or month 12 (*P* = 0.81). Fig 2 illustrates the distribution of patients achieving BCVA of not worse than 20/40 or not better than 20/200. No significant intergroup differences were observed at month 0 (*P* = 0.40), month 3 (*P* = 0.17), month 6 (*P* = 0.84), month 12 (*P* = 0.55), or month 18 (*P* = 0.84).

### Changes in BCVA over time

**Fig 3:**
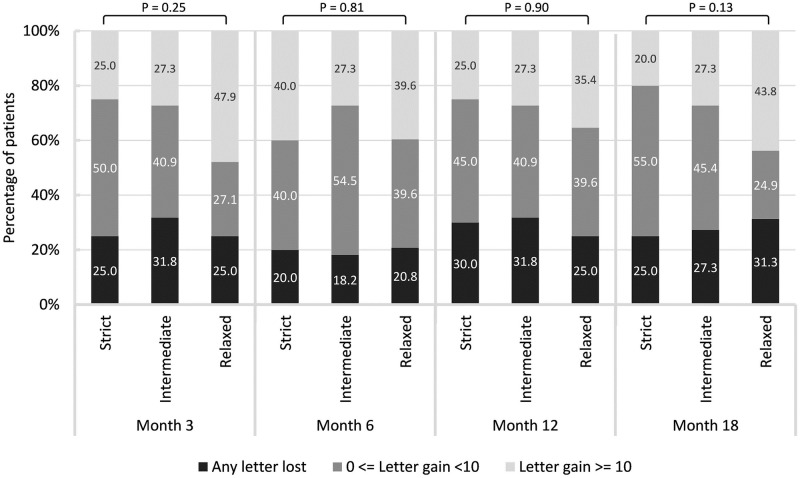
Changes in BCVA among the strict, intermediate, and relaxed groups, measured in terms of letters at various time points.

The overall average improvement in BCVA was 3.11±19.82 letters per patient at month 18. The strict group presented the least improvement in BCVA at month 18 (-1.12±17.98 letters), compared with the intermediate group (4.58±21.67 letters) and the relaxed group (4.21±19.84 letters; *P* = 0.56). Note however that these differences did not attain the level of significance. Similarly, we did not observe significant intergroup differences in BCVA improvement at month 3 (*P* = 0.12), month 6 (*P* = 0.76), or month 12 (*P* = 0.53). Fig 3 illustrates the distribution of patients gaining no less than 10 letters or losing any number of letters. No significant intergroup differences were observed at month 3 (*P* = 0.25), month 6 (*P* = 0.81), month 12 (*P* = 0.90), or month 18 (*P* = 0.13).

### Factors associated with changes in visual acuity

Table 5 presents regression analysis of factors associated with changes in visual acuity observed at 18 months. In univariate analysis, baseline visual acuity and presence of SRF at baseline were both inversely correlated with final visual gains (*P* < 0.01 and *P* = 0.02, respectively). After adjusting for age, sex, and baseline visual acuity, factors significantly correlated with poorer final visual gain included better baseline visual acuity, presence of SRF at baseline, and presence of IRF at month 18 (*P* < 0.01, *P* = 0.02, and *P* = 0.04, respectively). The factors presenting a non-significant correlation with final visual change included the following: age, sex, IRF at baseline, PED at baseline, pachychoroid at baseline, duration of retinal fluid retention, and duration of retinal fluid tolerance.

**Table 5:** Regression analysis of factors associated with changes in visual acuity at 18 months.

	Univariate coefficient	P value	Adjusted coefficient^¶^	P value
Age	-0.035	0.75	-0.051	0.61
Sex (male = 1)	-0.093	0.38	0.053	0.59
Baseline LogMAR	0.420	<0.01	0.416	<0.01
Baseline CRT, μm	0.139	0.19	0.006	0.96
SRF at baseline	-0.238	0.02	-0.230	0.02
IRF at baseline	-0.018	0.86	-0.180	0.09
PED at baseline	-0.065	0.54	-0.017	0.87
Pachychoroid at baseline	0.090	0.40	0.075	0.45
CRT at 18 months, μm	0.085	0.42	0.083	0.41
SRF at 18 months	-0.134	0.21	-0.094	0.35
IRF at 18 months	-0.174	0.10	-0.201	0.04
Total injection numbers	-0.142	0.18	-0.090	0.37
Total fluid duration, days	-0.073	0.49	-0.064	0.52
Total IRF duration, days	0.005	0.97	-0.090	0.37
Total IRF-tolerant duration, days	0.041	0.70	-0.029	0.77
Total SRF duration, days	-0.108	0.31	-0.062	0.53
Total SRF-tolerant duration, days	0.010	0.93	0.015	0.88

CRT, central retinal thickness; IRF, intraretinal fluid; LogMAR, logarithmic minimum angle of resolution; PED, pigment epithelial detachment; SRF, subretinal fluid.

^¶^AAge, sex, and baseline LogMAR were used for adjustment in multivariate analysis.

### Patients tolerating only SRF

There were 34 cases who tolerated only SRF during follow-up, i.e., no IRF was present at any visit when fluid was tolerated. In this group, the baseline LogMAR was 0.73±0.42, and the final logMAR at 18 months was 0.64±0.48. The mean visual gain was 4.64±13.83 letters. The baseline CRT was 289.9±78.9 μm, and the final CRT was 243.7±78.9 μm. The average change in CRT was -46.3±89.7 μm. Patients received an average of 6.18±1.93 injections. All of the aforementioned parameters were not significantly different from those in the strict group. The fluid tolerance ratio was 38.31±17.01%. The duration of retinal fluid divided by the total follow up time was 78.53±24.40%.

### Sensitivity analysis

Sensitivity analysis using cut off points of fluid tolerance ratio of 0, <20%, ≥20% and 0, <50%, ≥50% for strict, intermediate, relaxed group was done. In both conditions, despite the fact that the relaxed group presented a significant fewer total injection numbers and higher total duration of retinal fluids, the improvement in visual acuity was not significantly worse than that in the strict group.

## Discussion

In this real-world study, we examined nAMD patients presenting recurrent or persistent retinal fluid. We found that after 3 months of aflibercept loading, a more relaxed approach to dealing with retinal fluid (i.e., tolerating retinal fluid) produced similar results in terms of final visual outcome, in the setting of limited medical resources (mean 5.99 injections over 18 months).

The PrONTO study was an early investigation into the feasibility of individualized PRN strategy [10,11]. In that study, the mean visual gain was +9.3 letters resulting from a mean of 5.6 injections at year 1. The results were on par with those reported in the fixed-dose ANCHOR study (+11.3 letters with average 11.2 injections at year 1) with roughly half the number of injections [2]. In the CATT study, the mean visual gain in the PRN arm was +6.8 letters at 1 year following an average of 6.9 injections [12]. Those results indicate that similar visual gains can be achieved with fewer injections. It is important to note that in the PrONTO and CATT studies, the treatment endpoint was to resolve all retinal fluid.

A number of studies have investigated treatment outcomes in cases where retinal fluid was tolerated. The SUSTAIN study was a prospective study investigating a relaxed PRN strategy, in which a vision decrease of ≥ 5 letters or a CRT increase of ≥ 100 μm were adopted as criteria for retreatment [13]. After 1 year, the average improvement in vision was +3.6 letters following an average of 5.6 injections. In the FLUID study, the relaxed group adopted a T&E regimen tolerating 200 μm of SRF. After 1 year, the visual improvement was +4.3 letters following an average of 8.9 injections [7]. In a meta-analysis of 42 real-world studies, the mean visual improvement at 1 year was +3.5 letters following an average of 5.2 injections under the PRN regimen [14]. In the current study, the relaxed group presented an average visual improvement of +4.21 letters following an average of 4.92 injections at 18 months. Our results are in line with previous reports.

In studies on the prognostic effects of IRF and SRF, IRF has been linked to poor prognosis. In *post hoc* analysis of the VIEW1 and VIEW2 studies, the presence of IRF was associated with a mean decrease in 2.1 letters gained at 1 year [15]. A number of researchers have advocated more aggressive treatment for patients with IRF in order to maximize the effects on IRF resolution. In our study, although the presence of IRF at baseline was associated with poorer baseline visual acuity, no significant impact on the final visual gain was found. Instead, the presence of IRF at month 18 was associated with lesser final visual gain. This finding echoes with previous study [16]. It is possible that IRF affects visual acuity, and as the resolution of the IRF during treatment, the visual acuity also improves. It is the non-resolved IRF that hampers the visual improvement, not the initial presence of IRF.

On the other hand, *post hoc* analysis of the EXCITE, VIEW1, and VIEW2 studies revealed that patients with resistant SRF presented more pronounced visual gains than their counterparts without SRF [15,17]. Post hoc analysis of the CATT and HAROBR studies revealed that the presence of resistant SRF was associated with a lower risk of macular atrophy [9,18]. Based on these results, practitioners have come to view residual SRF as relatively benign form of nAMD. In the current study, we did not observe significant differences among the strict, intermediate, and relaxed groups in terms of macular atrophy incidence. This may be due to the relatively low total number of injections, the relatively short follow-up duration, or the presence of IRF in the fluid-tolerant groups. Regression analysis in the current study revealed that the presence of SRF at baseline had a negative impact on final visual gains. It is worth mentioning that our study excluded patients presenting dry retina at every follow-up visit after 3 monthly loading injections, i.e. those excellent treatment responders. It is possible that given the recurrence or persistence of retinal fluid during the course, the initial presence of SRF negatively affects visual improvement. Additionally, in the 34 cases in which only SRF was tolerated in our study, the visual gains were above the average of the rest of our cohort. This may also indicate that tolerating only SRF did not yield inferior visual outcomes.

Retinal fluid is not necessarily associated with underlying neovascular activity. A number of studies have reported on the presence of SRF or IRF without active vascular leakage [19,20]. It has been assumed that degenerative retinal fluids are the product of transudative or degenerative pathways, such as impaired RPE pumping and Muller cell damage [20,21]. Note that those effects are distinct from the exudative neovascular pathway mediated by VEGF. In cases of nAMD, degenerative and exudative fluid may both be present at the same time. Exudative fluid responds well to anti-VEGF therapy, indicating that degenerative retinal fluid may be refractory and persistent throughout the course of treatment [19]. However, in the current study, we did not differentiate between degenerative and exudative IRF during clinical visits. In the CATT study, retinal fluid was observed in 53.2% of the patients after receiving monthly doses of ranibizumab for a period of 1 year [12]. In the current study, 50.0% of the patients in the strict group presented with retinal fluid at 18 months. Clinicians should consider all of these mechanisms when treating residual retinal fluid following anti-VEGF injections, due to the fact that additional injections could actually exacerbate the process of degeneration. The 2-year CATT study revealed a link between monthly anti-VEGF injections and an elevated risk of geographic atrophy (compared to PRN injections) [22].

Numerous researchers have pointed out that anatomical improvements do not necessarily correspond to improvements in visual acuity. The FLUID study reported that intensive therapy aimed at achieving a completely dry macula cannot guarantee the desired visual gains [7]. In the VIEW 2 study, the proportion of patients who achieved dry retina was higher in the aflibercept arm (80.3%) than in the ranibizumab arm (60.4%); however, there was no significant difference between the two groups in terms of final VA gain [3]. In the current study, the strict group presented a more pronounced CRT reduction and higher proportion of patients achieving dry retina. However, the final visual acuity was similar between the three groups. Note that the baseline BCVA in the strict group tended to be better; therefore, the ceiling effect of visual acuity should also be considered, in which superior baseline vision resulted in less pronounced final visual gains, as reported in previous studies [23,24]. The regression analysis in our study also revealed a strong inverse correlation of initial visual acuity and final visual gain.

In the real-world, treatment imposes a considerable burden on the patient and the health system. Previous real-world studies have demonstrated the difficulties in adhering to a fixed-dose regimen without tolerance for any retinal fluid. One retrospective study in the UK reported a median of 2.0 visits during the maintenance phase (months 3–12) after the first anti-VEGF injection, which is lower than the quarterly follow-up [25]. In the SEVEN-UP study, the management of patients completing the ANCHOR and MARINA trial was at the discretion of the primary clinician. In that study, the mean number of injections during the mean 3.4-year follow-up period was only 6.8, with an average loss of 8.6 letters from baseline [26]. Researchers have also shown that reducing the number of injections can improve adherence to therapy, thereby reducing the incidence of treatment discontinuation [27]. In the current study, the relaxed fluid-tolerance group (mean 4.92 injections over 18 months) achieved visual outcomes similar to those in the strict fluid-intolerance group (mean 7.50 injections over 18 months) despite receiving significantly fewer injections. Note that mean clinical visits between the three groups were similar in our study. When implementing such relaxed approach, it is important to follow up continuously and prescribe treatment when necessary.

This study was subject to a number of limitations. First, this was a retrospective observational study with limited sample size. Patients were treated by different clinicians, leading to variations in treatment protocol and the frequency of clinical visits. Balancing baseline characteristics between each group was not perfect. Nonetheless, the heterogenic nature of this condition made it possible to investigate the impact of fluid tolerance on treatment outcomes. Moreover, compared to clinical trials, our data are more representative of the real-world conditions encountered in daily practice. Second, to investigate the effect of fluid tolerance, we excluded patients presenting dry retina at every follow-up visit after 3 monthly loading injections. This resulted in a different study cohort than a general nAMD population. The correlation between baseline characteristics and outcome measurements should be interpreted with caution. Third, the tolerance of retinal fluid in the treatment course is mixed with SRF only, IRF only, and combined presence of SRF and IRF. Proportions of patients with different fluid components, as well as the duration of tolerance of different fluid type were used to describe the condition. Fourth, the strict group in our study only had a mean of 7.50 injections over 18 months. Whether a more proactive treatment can result in significant difference in visual outcomes than the relaxed group (mean 4.92 injections over 18 months) is beyond the capability of our study. Finally, the mean follow-up duration was 18 months, such that the long-term effects of fluid-tolerant therapy on final outcomes remain unclear.

In conclusion, intravitreal aflibercept is effective in improving BCVA and CRT in nAMD patients presenting with recurrent or persistent retinal fluid after 3 months of aflibercept treatment. In our study, the relaxed group had 2.58 less injections (4.92 vs 7.50) and 7.1 more weeks of injection intervals (18.8 vs 11.7) compared with strict group. Despite the fact that the relaxed group presented a less pronounced reduction in CRT, the improvement in visual acuity was not significantly worse than that in the strict group. A more proactive injection frequency (>7.50 injections over 18 months) or a longer follow up time may be needed to achieve significant difference in visual outcomes. However, in the setting of real-world of limited medical resources, a fluid-tolerant approach can be implemented with comparative visual acuity gain. With fewer injections, the burden of medical care and the adherence of patient to the therapy might be improved.

## Supporting information

S1 Dataset (XLSX)
